# Sudachitin, polymethoxyflavone from *Citrus sudachi*, enhances antigen-specific cellular and humoral immune responses in BALB/c mice

**DOI:** 10.3164/jcbn.18-70

**Published:** 2018-12-05

**Authors:** Mami Mitani, Yuki Minatogawa, Akiko Nakamoto, Mariko Nakamoto, Emi Shuto, Yoshitaka Nii, Tohru Sakai

**Affiliations:** 1 Department of Public Health and Applied Nutrition, Institute of Biomedical Sciences, Tokushima University Graduate School, 3-18-15 Kuramoto-cho, Tokushima 770-8503, Japan; 2 Food and Biotechnology Division, Tokushima Prefectural Industrial Technology Center, 11-2 Saika-cho, Tokushima 770-8021, Japan

**Keywords:** sudachitin, *Citrus sudachi*, flavonoid, immune response, dendritic cells

## Abstract

Scdachitin is a polymethoxyflavone (5,7,4'-trihydroxy-6,8,3-trimethoxyflavone) that is found in the peel of *Citrus sudachi*. We examined the effect of sudachitin on immune response in ovalbumin-immunized BALB/c mice. Treatment with sudachitin increased ovalbumin-specific IL-4 and IL-10 productions. In addition, mice that received sudachitin showed higher levels of ovalbumin-specific IgE, IgG1 and IgG l production than did control mice. The antibody response to the thymus-independent antigen 2,4,6-trinitrophenyl Ficoll was not different in the control and sudachitin groups, suggesting that sudachitin does not directly stimulate antibody production. An *in vitro* study showed that treatment of sudachitin enhanced the ability of antigen presentation in bone marrow-derived dendritic cells. Furthermore, CD11c^+^ cells that had been treated with sudachitin showed increased expression of co-stimulatory molecules. The results indicate that sudachitin regulates immune function both *in vivo* and *in vitro*.

## Introduction

Exogenous antigens (Ags) are taken into Ag-presenting cells, such as dendritic cells, macrophages and B cells, and is resolved by various digestive enzymes including cathepsins in the process of transport from the endosome to lysosome or from the phagosome to phagolysosome. MHC class II molecules that are synthesized in the endoplasmic reticulum are covered with class II-associated invariant chain peptide (CLIP) and are trafficked through the Golgi apparatus. CLIP is eliminated in the endosome, and exogenous Ag-derived peptide binds to MHC class II. The complex is trafficked to the cell surface of Ag-presenting cells and presents that peptide to CD4^+^ cells.^(1–4)^ Specific Ag-recognizing naïve CD4^+^ cells differentiate into type 1 (Th1), Th2, Th17 and regulatory T cells and then regulate immune functions in the body.^(5–8)^

It is known that citrus peel contains various types of functional polyphenols. The polymethoxyflavoes possess several biological properties. Nobiletin (5,6,7,8,3',4'-hexamethoxyflavone) is one of the extensively studied polymethoxyflavone and has shown to be possess a wide range of pharmacological activities. *Citrus sudachi* is a special local product of Tokushima Prefecture in Japan. Sudachitin (5,7,4'-trihydroxy-6,8,3'-trimethoxyflavone) is a polymethxyflavone found in *Citrus sudachi* and is structurally similar to the nobiletin. We have evaluated the function of sudachitin and shown that treatment of sudachitin reduced gain of body weight in mice fed a high-fat diet. We also examined the mechanism and found that sudachitin increases sirtuin1, peroxisome proliferative-activated receptor gamma coactivator-1-α and uncoupling protein-1 gene expression levels, resulting in enhancement of energy expenditure.^(9)^

In addition to anti-obesity effect, it has been shown that sudachitin possesses anti-inflammatory action.^(10)^ However, effect of sudachitin on immune response *in vivo* was not known. In this study, we immunized mice with exogenous Ag ovalbumin (OVA) and examined the effect of sudachitin on OVA-specific immune responses in mice. Furthermore, to investigate immune regulatory mechanism by sudachitin, *in vitro* dendritic cell experiment was done.

## Materials

### Mice and diets

Female BALB/c mice (Japan SLC, Shizuoka, Japan) and C57BL/6 mice (Japan SLC, Shizuoka, Japan) were maintained under specific pathogen-free conditions with a 12-h light:dark cycle at 25 ± 2°C and 55 ± 10% relative humidity. Mice were given free access to water and food throughout the experiment. The mice were maintained on a control diet (no. D10012G; Research Diets Inc., NJ). All studies were performed in accordance with the ethical guidelines for animal experimentation by the Graduate School of Biomedical Sciences, Tokushima University, Japan and were approved by the institution review board of the animal ethics committee.

### Sudachitin treatment

Sudachitin (purity >98%) was provided by Tokushima Prefectural Industrial Technology Center (Tokushima, Japan). For oral administration, sudachitin was dissolved in dimethyl sulfoxide (DMSO). Sudachitin solution was prepared daily in 20 mmol/L of Na_2_CO_3_ aqueous solution (containing 0.1% DMSO). The mice were administered 200 µl of sudachitin solution containing 20 mg sudachitin/kg body for 35 days by gavage. Control mice were treated with a corresponding volume of DMSO with 200 µl of 20 mmol/L Na_2_CO_3_ aqueous solution (containing 0.1% DMSO).

### Immunization

Mice were immunized intraperitoneally with 500 µl of OVA solution containing 10 µg of OVA (Sigma Chemical Co., St. Louis, MO) absorbed in 2 mg of aluminium hydroxide gel adjuvant (Sigma-Aldrich Co., St. Louis, MO) on days 7 and 21 after the start of sudachitin administration.

In another experiment, mice were immunized with 25 µg of 2,4,6-trinitrophenyl (TNP)-Ficoll (Sigma Chemical Co.) in the same manner as that of OVA immunization.

### Cytokine production

Splenocytes (2.5 × 10^6^ cells/well) were stimulated with 400 µg/ml OVA in a 24-well flat-bottom plate at 37°C under 5% CO_2_ for 72 h. After the culture, culture supernatants were collected and stored at −30°C until used. Interferon (IFN)-γ, interleukin (IL)-2, IL-4 and IL-10 in the supernatants were quantified using a mouse IFN-γ (eBioscience, San Diego, CA), IL-4 (Biolegend, San Diego, CA), IL-10 (eBioscience) enzyme-linked immunosorbent assay (ELISA) kit according to the manufacturer’s instructions.

### Measurement of Ag-specific antibody (Ab)

 OVA-specific IgG, IgG1, IgG2a and IgE were measured by ELISA. OVA was dissolved with a coating buffer (50 mM carbonate-bicarbonate buffer, pH 9.6) at 10 µg/ml. Fifty µl of OVA solution was added to each well and incubated overnight at 4°C. After blocking with phosphate buffered saline (PBS) solution containing 1% bovine serum albumin (BSA), the diluted sera were incubated for 2 h. Subsequently, each well was washed three times with PBS containing 0.05% Tween 20. Incubation was performed for 2 h after addition of alkaline phosphatase (AP)-conjugated anti-mouse IgG, IgG1 or IgG2a Ab (Southern Biotechnology Associates, Inc., Birmingham, AL). After washing, 3,000-fold diluted AP-labeled streptavidin solution was added and incubated for 1 h at room temperature. *p*-Nitrophenyl phosphate was dissolved in 10% diethanolamine to prepare a 10% diethanolamine buffer, and the solution was added to each well. After color development, the reaction was stopped by addition of 3N NaOH and the absorbance was measured at a wavelength of 405 nm. For IgE Ab measurement, biotin-labeled anti-mouse IgE monoclonal (m) Ab (Becton Dickinson, San Diego, CA) was added to each well for 1 h at room temperature and then added HRP-labeled streptavidin for 1 h. For HRP-based detection, ABTS was used as substrate and reaction was measured at 450 nm.

TNP-BSA (Sigma Chemical Co.) was dissolved with a coating buffer at 10 µg/ml. TNP-BSA solution was added to each well and incubated overnight at 4°C. The following steps were the same as those used for OVA-specific Ab measurement except for the use of AP-conjugated anti-mouse IgG Ab and AP-conjugated anti-mouse IgM Ab.

### Suppression assay

CD4^+^CD25^+^ T cells and CD4^+^CD25^−^ T cells were purified from mouse spleens that had been treated with the vehicle or 20 mg/kg sudachitin using a CD4^+^CD25^+^ Regulatory T cell Isolation kit (Miltenyi Biotec Inc., Auburn, San Diego, CA) and Cell Sorter SH800 (SONY, Tokyo, Japan). To prepare accessory cells (ACs), splenocytes (1 × 10^8^ cells) were suspended in RPMI-1640 medium and incubated with ascites fluid containing anti-CD90 Ab for 20 min on ice. The cells were washed and T cells were eliminated using Goat Anti-Rat IgG microbeads and a MACS LD column (Miltenyi Biotec Inc.). These cells were incubated with 100 µg/ml of mitomycin for 45 min at 37°C and washed twice. CD4^+^CD25^+^ T cells were cultured in a 96-well plate with CD4^+^CD25^−^ T cells (2.5 × 10^4^ cells/50 µl), ACs (1 × 10^5^ cells/50 µl) and anti-mouse CD3 mAb (2 µg/ml) for 72 h at 37°C under 5% CO_2_. For the last 8 h of culture, 37 KBq of [^3^H]TdR was added to the wells, and the amount of [^3^H]TdR incorporated was measured by a scintillation counter (Aloka, Tokyo, Japan).

### *In vitro* dendritic cell experiment

Bone marrow (BM) cells were collected from the femurs of BALB/c mice. BM cells (1 × 10^6^ cells/ml) were cultured with GM-CSF (10 ng/ml) (eBioscience) for 7 days at 37°C under 5% CO_2_. Then the cells (2 × 10^6^ cells/ml) were cultured in a 24-well plate with lipopolysaccharide (LPS) from *E. coli* O11:B4 (100 ng/ml) (Sigma-Aldrich Co.) and sudachitin (0, 12.5, 25 or 50 µM) for 24 h.

For determination of Ag presentation ability, CD11c^+^ cells derived from BALB/c mice (3.3 × 10^3^ cells, 10^3^ cells, 3.3 × 10^2^ cells) were cultured with CD4^+^ T cells derived from C57BL/6 mice (1 × 10^5^ cells) in a 96-well round-bottom plate for 72 h at 37°C under 5% CO_2_. For the last 8 h of culture, 37 KBq of [^3^H]TdR was added to the wells, and the amount of [^3^H]TdR incorporated was measured by a scintillation counter (Aloka). In another experiment, anti-CD80 and/or anti-CD86 mAb were added into the culture to block CD80 and/or CD86-mediated interaction.

For analysis of the expression of co-stimulatory molecules, cells had been treated with LPS and/or sudachitin were washed and cultured in a 24-well plate for 24 h at 37°C under 5% CO_2_. The cells were stained with peridinin-chlorophyll-protein (PerCP)-conjugated anti-mouse CD40 mAb, phycoerythrin (PE)-conjugated anti-mouse CD80 mAb, allophecocyanin (APC)-conjugated anti-mouse CD86 mAb and fluoresceinisothiocyanate (FITC)-conjugated anti-mouse CD11c mAb for 30 min on ice in the dark. All Abs were purchased from eBioscience. Flow cytometric analysis was performed on Guava easyCyte using Guava Incyte software (Merck Millipore, Darmstadt, Germany).

### Statistics

Data were analyzed using Student’s *t* test. Data are expressed as means ± SD. Differences were considered significant at *p*<0.05.

## Results

### Sudachitin increases Ag-specific cellular and humoral immune responses

**Fig. 1:**
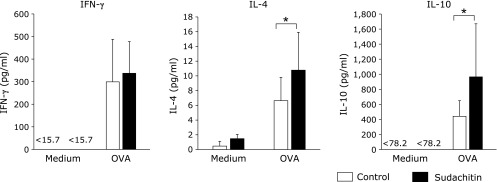
Sudachitin enhances antigen-specific cytokine productions in OVA-immunized mice. BALB/c mice were administered sudachitin by gavage and were immunized with OVA twice. Splenocytes from OVA-immunized mice were stimulated with OVA for 72 h. Concentrations of IFN-γ, IL-4 and IL-10 were determined by ELISA methods. Statistic difference was analyzed between control and sudachitin groups. *p<0.05.

**Fig. 2:**
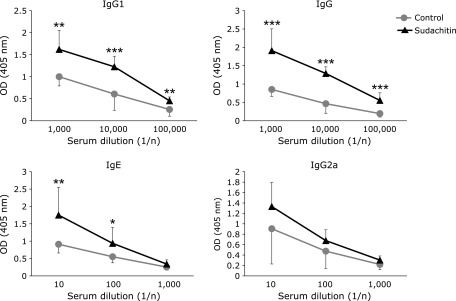
Sudachitin enhances antigen-specific antibody responses in OVA-immunized mice. BALB/c mice were administered sudachitin by gavage and were immunized with OVA twice. Levels of IgG1, IgG, IgE and IgG2a in diluted serum were measured by ELISA methods. Statistic difference was analyzed between control and sudachitin groups at same serum dilution level. *p<0.05, **p<0.01, ***p<0.001.

We investigated the effect of sudachitin on immune response. In previous study, we found that treatment of 5 mg/kg sudachitin attenuate body weight gain in C57BL/6 mice fed a high-fat diet.^(9)^ In preliminary experiment, we examined effect of sudachitin at dose 5, 10 and 20 mg/kg and found treatment of 20 mg/kg could obtain reproducible result. Mice were treated intraperitoneally with OVA and were administered sudachitin by gavage. We examined the *in vitro* proliferative responses of splenocytes by co-culture with OVA. The proliferative responses to OVA and anti-CD3 mAb were not significantly different in the control and sudachitin groups (Supplemental Fig. 1*). We also examined the levels of IFN-γ, IL-4 and IL-10 production. When splenocytes were stimulated with OVA, the levels of IL-4 and IL-10 production were increased in the sudachitin group compared to those in the control group, whereas the level of IFN-γ production was not significantly different (Fig. 1). In addition to cellular immune response, humoral immune response was investigated. The levels of OVA-specific IgG1, IgG and IgE were significantly increased in the sudachitin group compared with those in the control group, whereas the levels of OVA-specific IgG2a in the two groups were not different (Fig. 2). Flow cytometric analysis showed that the percentages of CD4^+^ cells, CD8^+^ cells, and CD44^low^ CD62L^high^ cells in CD4^+^ cells, the percentage of CD44^high^ CD62L^low^ cells in CD4^+^ cells and the percentage of CD25^+^ cells in CD4^+^ cells were not different between the control and sudachitin groups (Supplemental Fig. 2*).

### Sudachitin does not change TNP-specific IgM and IgG production

**Fig. 3:**
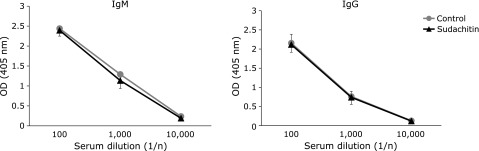
Sudachitin does not affect the Ab production against thymus-independent Ag. BALB/c mice were administered sudachitin by gavage and were immunized with TNP-Ficoll twice. Levels of IgM and IgG in diluted serum were measured by ELISA methods. Each value is the mean ± SD of 5 or 6 mice/group.

We found that sudachitin regulates OVA-specific immune responses. To examine the possibility that sudachitin directly stimulates B cells, resulting in increased Ab production, we further analyzed the effect of sudachitin on B cell response by using a thymus-independent Ag, TNP-Ficoll. Mice were treated with TNP-Ficoll and were administered sudachitin by gavage. The levels of TNP-specific IgM and IgG were not significantly different between the control and sudachitin groups (Fig. 3). These results suggest that the regulatory effect of sudachitin on Ag-specific immune responses is not a result of a direct effect on B cells.

### Sudachitin does not affect the suppressive activity of CD4^+^CD25^+^ cells

Treatment with sudachitin increased the Ag-specific immune response. We examined the possibility that sudachitin decreases the suppressive activity of CD4^+^CD25^+^ cells, resulting in enhancement of the immune responses. Suppressive activity of CD4^+^CD25^+^ cells was determined by the response of CD4^+^CD25^−^ cells to the stimulation of anti-CD3 mAb. No difference in the suppressive activity of CD4^+^CD25^+^ cells was observed at any ratio between the control and sudachitin groups (Suplemental Fig. 3*).

### Sudachitin enhances the ability of Ag presentation in dendritic cells *in vitro*

**Fig. 4:**
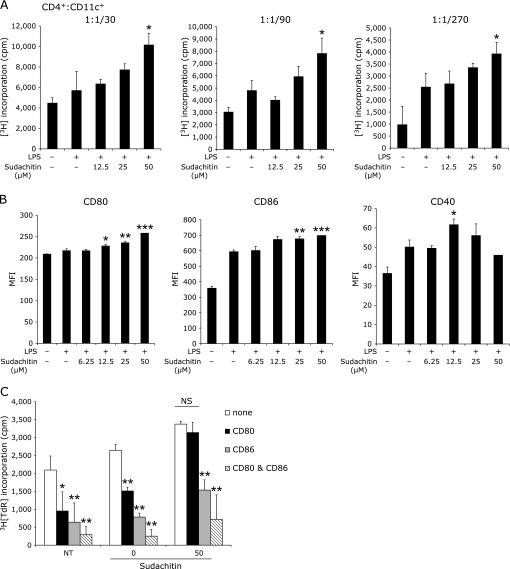
Sudachitin enhances the ability of antigen presentation in GM-CSF-induced dendritic cells *in vitro*. Bone marrow cells were cultured with GM-CSF for 7 days, and then the cells were cultured with LPS and/or sudachitin for 24 h. (A) CD11c^+^ cells were co-cultured with C57BL/6 mouse CD4^+^ cells at ratios of 1/30:1, 1/90:1 and 1/270:1 for 72 h. Antigen presenting ability was assessed by incorporation of [^3^H]TdR. (B) Dendritic cells were stained with FITC-conjugated anti-CD11c mAb, PE-conjugated anti-CD80 mAb, PerCP-conjugated CD40 mAb and APC-conjugated CD86 mAb. Expression levels of CD40, CD80 and CD86 in CD11c^+^ cells are shown as mean fluorescence intensity (MFI). (C) Evaluation of MLR was same as the method of Fig. 4A. The ratio of CD11c^+^ cells to CD4^+^ cells was 1:100. Dendritic cells were not treated or were treated with LPS or with LPS plus 50 µM sudachitin. NT means cells not treated with LPS. In this culture, anti-CD80 mAb (1 µg/ml) and/or anti-CD86 mAb (1 µg/m) were added. Each value is the mean ± SD. In Fig. 4A and B, statistic difference was analyzed between dendritic cells treated with LPS and dendritic cells treated with LPS plus sudachitin. In Fig. 4C, statistic difference was analyzed between dendritic cells treated with and without Ab. *p<0.05, **p<0.01, ***p<0.001.

Dendritic cells are major Ag-presenting cells that evoke an immune response. The effect of sudachitin on the function of dendritic cells was examined by the mixed lymphocyte reaction (MLR) method *in vitro*. Treatment with sudachitin increased MLR responses in a dose-dependent manner in LPS-stimulated CD11c^+^ cells, and a significant difference was observed with 50 µM sudachitin treatment compared to the control value (Fig. 4A). To address the mechanism, we first examined the expression of co-stimulatory molecules because these are crucial for exogenous Ag presentation. As shown in Fig. 4B, treatment with 12.5, 25 or 50 µM sudachitin and treatment with 25 or 50 µM sudachitin significantly increased the expression of CD80 and CD86 molecules, respectively. We examined the importance of up-regulation of co-stimulatory molecules by sudachitin using Abs specific for CD80 and CD86. The addition of either anti-CD80 or CD86 mAb inhibited the MLR response, and the addition of both dramatically inhibited the response. However, this inhibition observed in the sudachitin group was more resistant than those observed in the other groups (Fig. 4C).

## Discussion

It has been shown that plant-derived polyphenols have an impact on body function, especially immune function. Epigallocatechin-3-gallate (EGCG), which is the major constituent of green tea, sequestered the p65 subunit of transcription factor nuclear factor-κB in the cytoplasm and inhibited transcriptional activity of the nuclear factor-κB-driven promoter in toll-like receptor-mediated activation, resulting in reduction of inflammatory cytokine production.^(11)^ Kobori *et al.*^(12)^ examined the effect of the major polyphenol quercetin on adipose tissue inflammation in mice with diet-induced obesity. They showed that quercetin suppressed gene expression associated with the accumulation and activation of immune cells, including the accumulation of macrophages and lymphocytes in epididymal adipose tissue. In contrast to immune suppressive activity, enhancement of immune functions by natural occurring components has been shown in some studies. Tumors often inhibit T-cell function to escape from immune cell surveillance and cause immune dysfunction during tumor progression. Severe loss of both effector and memory T cells, downregulation of type 1 immune response and upregulation of type 2 immune response, and decreased proliferation of effector T cells were observed in mice bearing tumor cells. Treatment with curcumin prevented the loss of T cells, expanded central memory T cell/effector memory T cell populations, reversed type 2 immune bias and attenuated the tumor-induced inhibition of T cell proliferation in tumor-bearing mice.^(13)^ A beneficial effect of quercetin on recovery of irradiation-induced body damage has been shown. Radiation causes a decrease in immune function in mice, but treatment with quercetin after irradiation reversed immune function including IL-1 and IL-6 production.^(14)^ In the present study, sudachitin increased the levels of OVA-specific cytokine production and OVA-specific IgG, IgG1 and IgE responses (Fig. 1 and 2). To our knowledge, this is the first study showing enhancement of both cellular and humoral immune responses by general flavonoids *in vivo* in mice. Our results indicate that sudachitin might be useful for preventing immune dysfunction caused by cancer, irradiation therapy and aging.

The structure of sudachitin resembles that of nobiletin (3',4',5,6,7,8-hexathoxflavon). Nobiletin is a polyphenol and its function has been studied extensively. It has been shown that nobiletin has exhibit biological effects including anti-inflammatory, anti-tumor and neuroprotective effects.^(15–20)^ Lin *et al.*^(16)^ reported that nobiletin decreased LPS-induced production of prostaglandin E2 and proinflammatory cytokines including IL-1α, IL-1β, TNF-α and IL-6 in mouse J774 macrophage cells. Sudachitin also has anti-inflammatory properties. Yuasa *et al.*^(10)^ examined the effects of the polymethoxyflavones sudachitin and nobiletin on LPS-induced inflammatory responses in mouse macrophage-like RAW264 cells. Both sudachitin and nobiletin suppressed LPS-induced TNF-α production, but the inhibitory effect of sudachitin was greater than that of nobiletin. Thus, it is worth examining effect of sudachitin on anti-tumor immunity and neuroprotective effect. In addition to the anti-inflammatory action of nobiletin, it has been shown that nobiletin has an anti-obese effects.^(20)^ We have also shown that sudachitin attenuates gain of body weight and improves lipid metabolism in mice fed a high-fat diet.^(9)^ In recent study, sudachitin decreased activation of mitogen-activated protein kinase and reactive oxygen species production evoked by receptor of nuclear factor-κB ligand in osteoclast linage cells. This finding suggests that sudachitin is a useful agent for the treatment of anti-inflammatory bone destruction.^(21)^

Regulatory T (Treg) cells play roles in the maintenance of immunological unresponsiveness and in suppression of excessive immune responses. In this study, we observed enhancement of Ag-specific cellular and humoral immune responses. To address the mechanisms, we focused on Treg cells, which are major immune suppressive cells, because deletion of Treg cells by anti-CD25 mAb has been shown to enhance protective immunity against tumors and pathogens.^(22,23)^ Treg cell-mediated attenuation of autoimmune arthritis has been shown in EGCG. EGCG decreased the arthritis index and showed protective effects against joint destruction in an IL-1α knock out arthritis model. The proportion of Foxp3^+^ Treg cells was increased in the spleens of mice treated with EGCG. EGCG suppressed the activation of mammalian target of rapamycin and hypoxia inducible factor-1α, which is considered to be a metabolic check point of Treg cell differentiation.^(24)^ We examined the ratio of Treg cells in the spleen and their suppressive activity, but difference was not observed (Supplemental Fig. 3*).

Although we found a unique biological property of sudachitin, this study has some limitations. We could not ascertain directly whether the Ag-presentation ability of dendritic cells is increased *in vivo*. We determined the expression of MHC class II and co-stimulatory molecules in splenic CD11c^+^ cells by flow cytometric analysis, but we did not find a significant difference between the control and sudachitin groups. Furthermore, the value of MLR using CD11c^+^ cells from splenocytes of mice treated with sudachitin was not different from the value of MLR using CD11c^+^ cells from splenocytes of control mice (data not shown). In an *in vitro* experiment, we used sudachitin at a dose of 50 µM. In our preliminary experiment, treatment with 50 µM sudachitin did not affect cell viability as estimated by the propium iodide/annexin V staining method (data not shown).

We found that sudatichin not only affects mitochondrial energy regulation^(9)^ but also regulates immune function i*n vivo*. Although the mechanism by which sudachitin enhances Ag-specific immune response can be partly explained by regulation of dendritic cell function based on the results of our *in vitro* experiment, the precise mechanism remains to be determined. Further studies on the regulatory mechanisms of sudachitin are required for expanded clinical applications.

## Abbreviations

Ab: antibody
Ag: antigen
AP: alkaline phosphatase
APC: allophecocyanin
BM: bone marrow
BSA: bovine serum albumin
CLIP: class II-associated invariant chain peptide
DMSO: dimethyl sulfoxide
ELISA: enzyme-linked immunosorbent assay
FITC: fluoresceinisothiocyanate
IFN: interferon
IL: interleukin
LPS: lipopolisaccharide
m: monoclonal
MLR: mixed lymphocyte reaction
OVA: ovalbumin
PerCP: peridinin-chlorophyll-protein
PBS: phosphate buffered saline
PE: phycoerythrin
TNP: 2,4,6-trinitrophenyl
Treg: regulatory T
