# Supplementary material for: Sudachitin, polymethoxyflavone from Citrus sudachi, enhances antigen-specific cellular and humoral immune responses in BALB/c mice
Source: J Clin Biochem Nutr. 2018 Dec 5;64(2):158–63. doi: 10.3164/jcbn.18-70 (PMC6436041; doi:10.3164/jcbn.18-70)
Supplement: Supplemental Figure 1 [file jcbn18-70sf01.pdf]

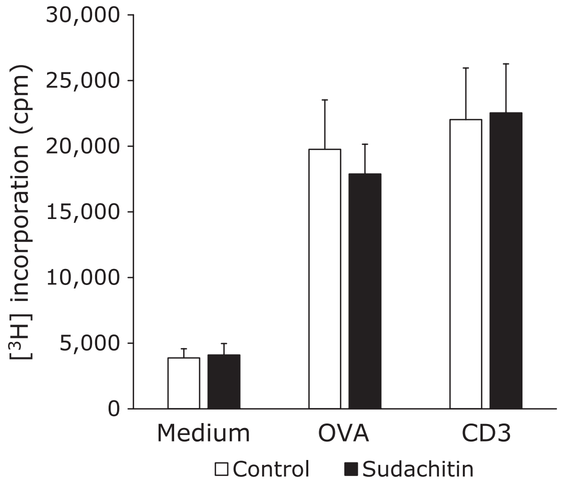

Supplemental Fig. 1. Proliferative response to OVA in mice immunized with OVA and treated with sudachitin. Splenocytes were cultured in PRMI-1640 medium supplemented with 10% fetal bovine serum, 50 μmol/L 2-mercaptoethanol, 100 μg/ml streptomycin, and 100 U/ml penicillin. Splenocytes (5×10^5 cells/well) were stimulated with 400 μg/ml OVA or with plate-bound anti-CD3 monoclonal (m)Ab and anti-mouse CD28 (coated overnight at 1 μg/ml) in a 96-well flat-bottom plate at 37°C under 5% CO_2 for 72 h. For the last 8 h of culture, 37 KBq of [^{3}H]thymidine deoxyribose (TdR) was added to the wells, and the amount of [^{3}H]TdR incorporated was measured by a scintillation counter.
