# Supplementary material for: Sudachitin, polymethoxyflavone from Citrus sudachi, enhances antigen-specific cellular and humoral immune responses in BALB/c mice
Source: J Clin Biochem Nutr. 2018 Dec 5;64(2):158–63. doi: 10.3164/jcbn.18-70 (PMC6436041; doi:10.3164/jcbn.18-70)
Supplement: Supplemental Figure 2 [file jcbn18-70sf02.pdf]

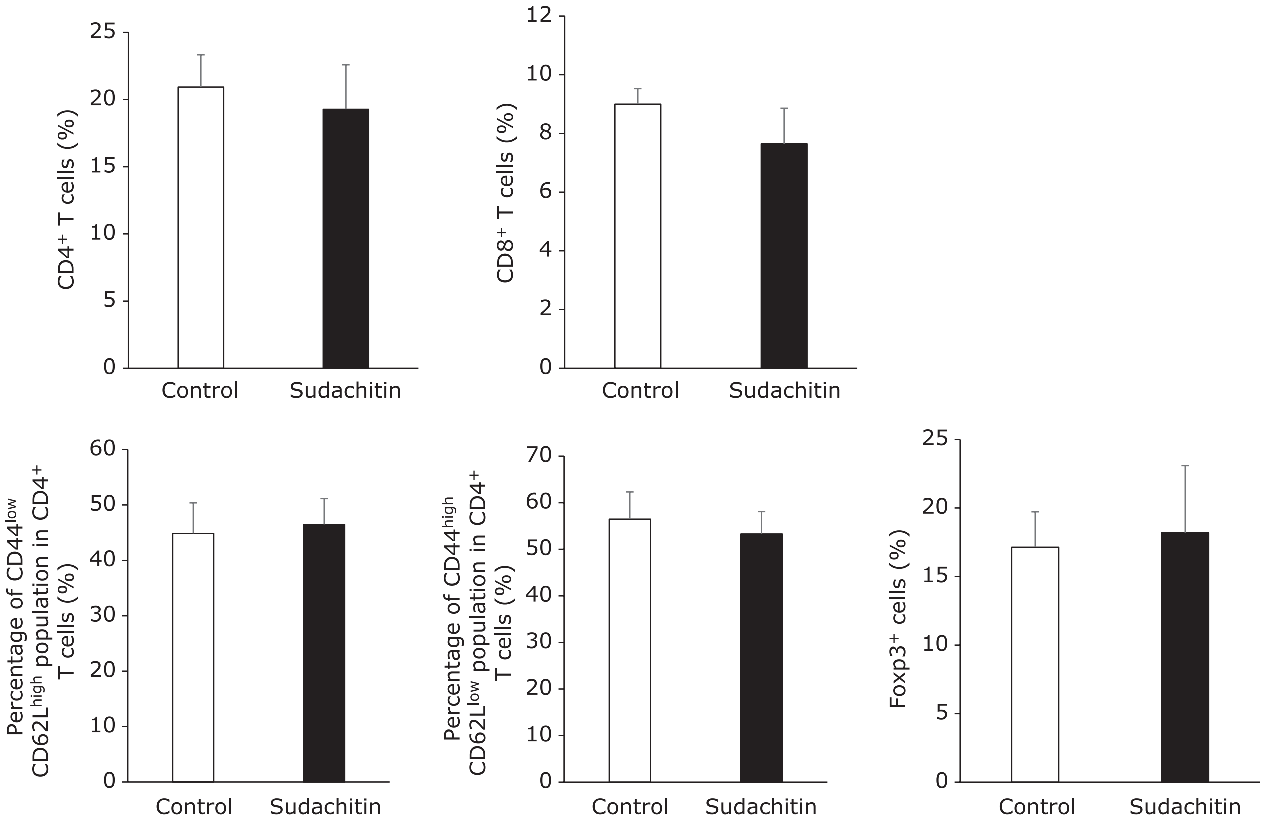

Supplemental Fig. 2. Lymphocyte subset analysis in mice immunized with OVA and treated with sudachitin. For lymphocyte subset analysis, splenocytes were stained with PE-conjugated anti-mouse CD4 mAb, FITC-conjugated anti-mouse CD8α mAb, PerCP-conjugated anti-mouse CD62L mAb and APC-conjugated anti-mouse CD44 mAb for 30 min on ice in the dark. Flow cytometric analysis was performed on Guava easyCyte using Guava Incyte software (Merck Millipore, Darmstadt, Germany). For foxp3 analysis, splenocytes were stained with FITC-conjugated anti-mouse CD4 mAb and PE-conjugated anti-mouse CD25 mAb for 30 min on ice in the dark. The cells were washed twice and stained for foxp3 using the FOXP3 Fix/Perm Buffer Set (BioLegend).
