# Supplementary material for: Sudachitin, polymethoxyflavone from Citrus sudachi, enhances antigen-specific cellular and humoral immune responses in BALB/c mice
Source: J Clin Biochem Nutr. 2018 Dec 5;64(2):158–63. doi: 10.3164/jcbn.18-70 (PMC6436041; doi:10.3164/jcbn.18-70)
Supplement: Supplemental Figure 3 [file jcbn18-70sf03.pdf]

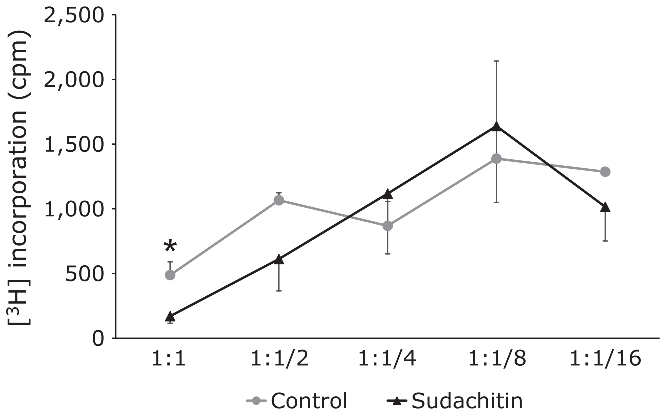

Supplemental Fig. 3. Suppresive function of CD4$^+$CD25$^+$ T cells from mice treated with sudachitin. CD4$^+$CD25$^+$ T cells and CD4$^+$CD25$^-$ T cells were purified from mouse spleens that had been treated with the vehicle or 20 mg/kg sudachitin using a CD4$^+$CD25$^+$ Regulatory T cell Isolation kit (Miltenyi Biotec Inc., Auburn, CA) and Cell Sorter SH800 (SONY, Tokyo, Japan). To prepare accessory cells (ACs), splenocytes (1 × 10^8 cells) were suspended in RPMI-1640 medium and incubated with ascites fluid containing anti-CD90 Ab for 20 min on ice. The cells were washed and T cells were eliminated using Goat Anti-Rat IgG microbeads and a MACS LD column (Miltenyi Biotec Inc.). These cells were incubated with 100 μg/ml of mitomycin for 45 min at 37°C and washed twice. CD4$^+$CD25$^+$ T cells were cultured in a 96-well plate with CD4$^+$CD25$^-$ T cells (2.5 × 10^4 cells/50 μl), ACs (1 × 10^5 cells/50 μl) and anti-mouse CD3 mAb (2 μg/ml) for 72 h at 37°C under 5% CO_2. For the last 8 h of culture, 37 KBq of [^{3}H]TdR was added to the wells, and the amount of [^{3}H]TdR incorporated was measured by a scintillation counter.
